# REGEN‐COV antibody combination in patients with lymphoproliferative malignancies and SARS‐CoV‐2 infection

**DOI:** 10.1002/jha2.403

**Published:** 2022-02-23

**Authors:** Yotam Bronstein, Irit Avivi, Yael C Cohen, Eugene Feigin, Chava Perry, Yair Herishanu

**Affiliations:** ^1^ Department of Hematology Sackler Faculty of Medicine Tel Aviv Sourasky Medical Center Tel Aviv University Tel Aviv Israel; ^2^ Internal Medicine Department D Sackler Faculty of Medicine Tel Aviv Sourasky Medical Center Tel Aviv University Tel Aviv Israel

**Keywords:** Covid‐19, lymphoproliferative malignancies, REGEN‐COV, SARS‐Cov‐2

## Abstract

Patients with lymphoproliferative diseases are at high risk for SARS‐CoV‐2‐related complications and mortality. The role of casirivimab and imdevimab (REGEN‐COV), a neutralizing antibody cocktail, to treat immunocompromised hemato‐oncological patients with SARS‐CoV‐2 disease 2019 (Covid‐19) remains unknown. Here, we present our clinical experience on the outcome of 15 hematological patients treated with REGEN‐COV for SARS‐CoV‐2 infection. Most patients failed to respond or achieved low antibody titer after 2–3 doses of BNT162b2 mRNA vaccine. All patients experienced clinical improvement with no mortality within a median follow‐up of 70 days. In conclusion, early administration of REGEN‐COV to high‐risk hematological patients may prevent clinical deterioration and mortality from SARS‐CoV‐2 infection. The effectiveness of neutralizing antibodies may vary depending on the virus variants and in particular with the omicron variant (B.1.1.529).

## 1 INTRODUCTION

Patients with lymphoproliferative malignancies are at high risk of developing severe Covid‐19 disease and death [1]. Several studies have shown that despite the high antibody responses to Covid‐19 vaccines in the general population, responses to vaccination in patients with hematological malignancies, especially in those treated with anti‐CD20 based therapies or Bruton's tyrosine kinase inhibitors (BTKis), are relatively low [2, 3]. Therefore, a key challenge is how effectively to treat immunocompromised patients with SARS‐CoV‐2 infection and how to prevent clinical deterioration.

Casirivimab and imdevimab (REGEN‐COV) is a neutralizing antibody cocktail [4], explicitly directed against the spike protein of SARS‐CoV‐2. In November 2020, REGEN‐COV received an emergency use authorization (EUA) from the US Food & Drug Administration (FDA) [5] for the treatment of mild to moderate COVID‐19 in adults and pediatric patients who are at high risk for progression to severe COVID‐19, including hospitalization or death [6, 7]. However, there is a lack of data regarding the clinical efficacy of REGEN‐COV in hemato‐oncological patients. Herein, we report the outcome of 15 patients with hematological malignancies and SARS‐CoV‐2 infection treated with REGEN‐COV.

## 2 METHODS

### 2.1 Study design and patients

We conducted a retrospective, single center study of all consecutive hematological patients who were diagnosed with Covid‐19 and were treated with a single infusion of casirivimab (600 mg) and imdevimab (600 mg) (REGEN‐COV, ROCHE). According to our hospital policy, immunocompromised patients diagnosed with Covid‐19 within the last 10 days since initiation of symptoms were eligible to receive REGEN‐COV. SARS‐CoV‐2 infection was confirmed by reverse transcription‐polymerase chain reaction (RT‐PCR) testing. Covid‐19 severity was graded according to the Israeli ministry of health protocol: mild disease was defined in the presence of fever and/or cough and/or, significant weakness, a moderate disease was defined in the presence of Covid‐19‐related pneumonia (confirmed radiologically), and severe disease was determined if respiratory rate was greater than 30 breaths per minute and/or oxygen saturation ≤93% at room air or PaO_2_/FiO_2_ ratio <300 [8]. Data were extracted from the patients' medical records and included baseline demographics, disease‐related and treatment‐related characteristics, Covid‐19 vaccination status, anti‐SARS‐CoV‐2 IgG titers, complete blood count at admission to the hospital and clinical data on Covid‐19 infection, treatment, and outcome. The study was approved by the local institutional Helsinki ethics committee.

## 3 RESULTS

### 3.1 Patient characteristics

From July through October 2021, a total of 15 patients with hematological malignancies were included in this study (patient baseline demographic and disease characteristics are summarized in Table 1). The median age of the patients was 60 years (range 28–77) and 60% (*n* = 9) were males. The majority of patients were diagnosed with B‐cell non‐Hodgkin lymphoma (60%, 9/15), followed by multiple myeloma (20%, 3/15), chronic lymphocytic leukemia (13%, 2/15) and T‐cell lymphoma (7%, 1/15). Among all patients, 67% (*n* = 10) were actively treated at the time of SARS‐CoV‐2 infection (3 with anti‐CD20 based therapy, 2 with BTKis, 3 with immunotherapy, 1 with anti‐CD38 antibody, and 1 shortly after an autologous hematopoietic stem cell transplantation [HSCT]). The other five patients (33%) have been previously treated (3 with anti‐CD20 therapies, 1 after chimeric antigen receptor T cell therapy, and 1 after autologous HSCT), with a median time of 18 months (range 12–32) from end of treatment to the time of diagnosis of SARS‐CoV‐2 infection.

**TABLE 1:** Patients' baseline demographic and disease characteristics

Patient number	Gender	Age (years)	Hematological malignancy	Treatment status at the time of SARS‐CoV‐2 infection	Last hematological treatment
1	M	49	DLBCL	On therapy in CR	R‐CHOP
2	M	74	DLBCL	Off therapy in CR	CAR‐T, 10/2019
3	F	37	FL	Off therapy in CR	Bendamustine, obinutuzumab, 12/2018
4	M	22	Burkitt lymphoma	Off therapy in CR	R‐CODOX‐M IVAC, 05/2020
5	F	74	CLL	Off therapy in PR	Obinutuzumab, 03/2020
6	F	77	CLL	On therapy in CR	Ibrutinib
7	M	60	Enteropathyassociated T‐cell lymphoma	Off therapy in CR	Autologous HSCT, 08/2020
8	F	71	FL	On therapy in PR	Revlimid and rituximab
9	M	41	Mantle cell lymphoma	On therapy in PR	Autologous HSCT
10	M	75	DLBCL	On therapy—active disease	R‐CHOP
11	M	56	Mantle cell lymphoma	On therapy in CR	Ibrutinib
12	M	51	MM	On therapy—active disease	Carfilzomib, daratumumab, and dexamethasone
13	F	28	PMBCL	On therapy—active disease	Brentuximab vedotin and nivolumab
14	M	74	MM	On therapy—active disease	Belantamab mafodotin
15	F	60	MM	On therapy—active disease	CAR‐T

Abbreviations: DBCL = diffuse large B‐cell lymphoma, FL = follicular lymphoma, CLL = chronic lymphocytic leukemia, MM = multiple myeloma, PMBCL = primary mediastinal large B‐cell lymphoma, CR = complete response, PR = partial response, R‐CHOP = rituximab, cyclophosphamide, doxorubicin, vincristine and prednisone, CAR‐T = chimeric antigen receptor therapy, R‐CODOX‐M IVAC = cyclophosphamide, vincristine, doxorubicin, high‐dose methotrexate/ifosfamide, etoposide, high‐dose cytarabine, HSCT = hematopoietic stem‐cell transplantation.

All patients received at least 2 doses of BNT162b2 mRNA Covid‐19 vaccine, administered 4–8 months prior to SARS‐CoV‐2 infection (median, 7 months). Nine patients (60%) received 2 doses of vaccine and six (40%) received 3 doses (Table 2). A total of 10 patients (67%) were serologically tested for SARS‐CoV‐2 Spike protein IgG levels at the emergency room (ER) before receiving REGEN‐COV. Seven (70%) were found to be seronegative, and three (30%) seropositive (one seropositive patient had a very low antibody level: 1.88, positive >1 u/ml). The median time from initiation of Covid‐19 related symptoms/diagnosis and the administration of REGEN‐COV was 4 days (range 1–12) and 2 days (range 0–12), respectively.

**TABLE 2:** Patients' Covid‐19 vaccination status and clinical parameters of SARS‐CoV‐2 infection

Patient number	Vaccination status	Time of SARS‐CoV‐2 infection from last vaccine	Absolute lymphocyte count on admission (10^9^/L)	SARS‐CoV‐2 spike protein IgG serology test on admission	Time of REGEN‐COV treatment from SARS‐CoV‐2 infection (day)	Covid‐19 severity in ER	Clinical follow‐up	Covid‐19 severity in discharge
1	Two doses	8 months	0.3	N/A*	1	Mild	Discharged from ER	Mild
2	Two doses	4 months	0.4	Seronegative	12	Severe	Hospitalized for 5 days	Severe
3	Two doses	6 months	1.5	Seronegative	2	Moderate	Hospitalized for 5 days	Moderate
4	Two doses	6 months	1.2	Seropositive (very low titer)	0**	Mild	Discharged from ER	Mild
5	Booster	1.5 months	1.1	Seropositive	9	Severe	Hospitalized for 12 days	Severe
6	Booster	3 weeks	1.4	Seronegative	3	Mild	Discharged from ER.	Mild
7	Two doses	7 months	0.6	N/A	2	Mild	Discharged from ER	Mild
8	Booster	3 weeks	0.8	N/A	1	Mild	Discharged from ER	Mild
9	Two doses	8 months	1.1	N/A	4	Mild	Discharged from ER	Mild
10	Two doses	8 months	0.3	Seropositive	0	Severe	Hospitalized for 4 days	Moderate
11	Booster	2 days	0.9	Seronegative	3	Mild	Discharged from ER	Mild
12	Two doses	7 months	0.4	Seronegative	0	Severe	Hospitalized for 3 days	Mild
13	Two doses	5 months	0.6	Seronegative	4	Mild	Discharged from ER	Mild
14	Booster	2 weeks	1.1	Seronegative	1	Mild	Hospitalized for 3 days	Mild
15	Booster	1.5 months	0	N/A	6	Severe	Hospitalized for 4 days	Severe

* N/A, the data is not available.

** Day 0 = day on admission.

Abbreviation: ER = emergency department.

### 3.2 Covid‐19‐related symptoms and outcome

Thirteen patients (87%) suffered from symptoms prior to admission to the ER. Median time from the onset of symptoms until a positive RT‐PCR for SARS‐CoV‐2 test was 1 day (range 1–12). The most common symptoms were fever (77%, 10/13), followed by cough (61%, 8/13), dyspnea (31%, 4/13), and weakness (31%, 4/13). Most of the patients (93%, 14/15) presented with lymphopenia (absolute lymphocytic count [ALC]: median of 0.6 × 10^9^/L; range 0–1.4 × 10^9^/L). Seven of the 13 symptomatic patients were considered as having a mild Covid‐19 infection and 6 patients had a moderated to severe disease (1 moderate, 5 severe) requiring hospitalization. All hospitalized patients were discharged within 3–12 days, with no clinically significant short or long‐term complications. Two patients received REGEN‐COV, despite being asymptomatic, given that they were at high risk of progression to a serious illness (both were severely lymphopenic; one had been recently treated with anti‐CD20‐based therapy and another patient after HSCT 1‐year prior to SARS‐CoV‐2 infection). After a median follow‐up of 70 days (range 30–110) for the entire cohort, none of the patients had clinical deterioration and all patients were alive.

## 4 DISCUSSION

Patients with lymphoproliferative malignancies are at high risk of mortality from Covid‐19, especially if recently treated with B‐cell depleting agents [1]. Covid‐19 vaccines provide immunity to some degree in patients with hematological malignancies. While patients treated with anti‐CD20 antibodies, BTK‐inhibitors or anti‐CD38 therapy [9], often fail to respond and are considered to be at high risk for a severe Covid‐19 disease [2, 3, 10].

Here we report the outcome of 15 high‐risk patients with lymphoproliferative malignancies who developed SARS‐CoV‐2 infection and were treated REGEN‐COV. According to our findings, administration of REGEN‐COV to patients, mostly presenting with mild Covid‐19 disease, resulted with favorable clinical outcomes while none of the patients has clinically deteriorated (requiring high‐flow oxygen, intubation, or additional Covid‐19 therapy).

Recent clinical studies in immunocompetent subjects suggested that anti‐SARS‐CoV‐2 neutralizing monoclonal antibodies are particularity effective in patients with mild to moderate Covid‐19 [11, 12]. Given our experience that patients with severe Covid‐19 (all seronegative) have improved with REGEN‐COV and none of them died, suggests that anti‐SARS‐CoV‐2 neutralizing antibodies might have a role also in immunocompromised patients with a more severe disease. Furthermore, a recent study [13] demonstrated that among hospitalized patients with Covid‐19, REGEN‐COV treatment reduced 28‐day mortality among patients who were seronegative in baseline.

According to our local treatment protocol, anti‐SARS‐CoV‐2 neutralizing antibody cocktails are given early, up to 10 days, after the development of symptoms, aiming to prevent clinical deterioration. Nevertheless, in immunocompromised patients, especially in those treated with anti‐CD20 antibodies, our local protocol allows treatment with REGEN‐COV after more than 10 days from onset of symptoms, after consulting with an infectious disease specialist. Accordingly, two patients received REGEN‐COV after more than 10 days (range 11–12) at the time of admission to the ER. Both patients were discharged from hospitalization after 5–12 days in stable condition without clinical deterioration. Although this clinical experience is limited, it provides a hint that REGEN‐COV administration after a longer period of time from the onset of symptoms may still be beneficial in some immunocompromised patients.

In summary, in this retrospective study, we show that early administration of REGEN‐COV to high‐risk hematological patients infected with the SARS‐CoV‐2 prevented clinical deterioration and mortality. Our cohort included patients treated with REGEN‐COV prior to the outbreak of the omicron variant, which for this highly mutated variant REGEN‐COV is expected to be less effective. Larger prospective studies investigating the role of REGEN‐COV in treating high‐risk hematological patients, infected with Covid‐19 or preventing infection, against different SARS‐CoV‐2 variants (Delta and Omicron) (B.1.617.2, B.1.1.529, respectively), are warranted.

## AUTHOR CONTRIBUTIONS

IA, YH, YC, EF, and CP provided clinical data for the patients. YB and IA wrote the manuscript. YH revised the manuscript. All authors reviewed and commented on the revised manuscript before submission.

## DECLARATION OF COMPETING INTEREST

The authors have no competing interests.

## References

[1] Vijenthira A , Gong IY , Fox TA , Booth S , Cook G , Fattizzo B , et al. Outcomes of patients with hematologic malignancies and COVID‐19: a systematic review and meta‐analysis of 3377 patients. Blood. 2020;136(25):2881–92. 33113551 10.1182/blood.2020008824 PMC7746126

[2] Herzog Tzarfati K , Gutwein O , Apel A , Rahimi‐Levene N , Sadovnik M , Harel L , et al. BNT162b2 COVID‐19 vaccine is significantly less effective in patients with hematologic malignancies. Am J Hematol. 2021;96(10):1195–203. 34185336 10.1002/ajh.26284 PMC8420332

[3] Mittelman M , Magen O , Barda N , Dagan N , Oster HS , Leader A , et al. Effectiveness of the BNT162b2mRNA Covid‐19 vaccine in patients with hematological neoplasms. Blood. 2021. Online ahead of print. 10.1182/blood.2021013768 PMC8526123 34662390

[4] Weinreich DM , Sivapalasingam S , Norton T , Ali S , Gao H , Bhore R , et al. REGN‐COV2, a neutralizing antibody cocktail, in outpatients with Covid‐19. N Engl J Med. 2021;384(3):238‐51. 33332778 10.1056/NEJMoa2035002 PMC7781102

[5] Coronavirus (COVID‐19) update: FDA authorizes monoclonal antibodies for treatment of COVID‐19. November 21, 2020. https://www.fda.gov/news-events/press-announcements/coronavirus-covid-19-update-fda-authorizes-monoclonal-antibodiestreatment-covid-19

[6] Razonable RR , Pawlowski C , O'Horo JC , Arndt LL , Arndt R , Bierle DM , et al. Casirivimab‐Imdevimab treatment is associated with reduced rates of hospitalization among high‐risk patients with mild to moderate coronavirus disease‐19. EClinicalMedicine. 2021;40:101102. 34485873 10.1016/j.eclinm.2021.101102 PMC8404031

[7] Weinreich DM , Sivapalasingam S , Norton T , Ali S , Gao H , Bhore R , et al. REGEN‐COV antibody combination and outcomes in outpatients with Covid‐19. N Engl J Med. 2021;385(23):e81. 34587383 10.1056/NEJMoa2108163 PMC8522800

[8] Israeli ministry of health protocol for COVID‐19 disease severity. July 12, 2020. https://www.gov.il/BlobFolder/reports/mr-294754420/he/files_publications_corona_mr-294754420.pdf

[9] Pimpinelli F , Marchesi F , Piaggio G , Giannarelli D , Papa E , Falcucci P , et al. Fifth‐week immunogenicity and safety of anti‐SARS‐CoV‐2 BNT162b2 vaccine in patients with multiple myeloma and myeloproliferative malignancies on active treatment: preliminary data from a single institution. J Hematol Oncol. 2021;14(1):81. 34001183 10.1186/s13045-021-01090-6 PMC8128283

[10] Chavez‐MacGregor M , Lei X , Zhao H , Scheet P , Giordano SH . Evaluation of COVID‐19 mortality and adverse outcomes in US patients with or without cancer. JAMA Oncol. 2021;8(1):69–78. 10.1001/jamaoncol.2021.5148 PMC8554684 34709356

[11] New analyses of two AZD7442 COVID‐19 Phase III trials in high‐risk populations confirm robust efficacy and long‐term prevention. Novemeber 18, 2021. https://www.astrazeneca.com/media-centre/press-releases/2021/new-analyses-of-two-azd7442-covid-19-phase-iii-trials-in-highrisk-populations-confirm-robust-efficacy-and-long-term-prevention.html

[12] Ganesh R , Pawlowski CF , O'Horo JC , Arndt LL , Arndt RF , Bell SJ , et al. Intravenous bamlanivimab use associates with reduced hospitalization in high‐risk patients with mild to moderate COVID‐19. J Clin Invest. 2021;131(19). 10.1172/JCI151697 PMC8483756 34411003

[13] Group RC , et al. Casirivimab and imdevimab in patients admitted to hospital with COVID‐19 (RECOVERY): a randomised, controlled, open‐label, platform trial. The Lancet. 2022. 399(10325):665–676. 10.1016/S0140-6736(22)00163-5. PMC8830904 35151397

